# Significance of intracellular localization of survivin in cervical squamous cell lesions: Correlation with disease progression

**DOI:** 10.3892/ol.2014.1948

**Published:** 2014-03-07

**Authors:** SOO-AH KIM, RAN HONG

**Affiliations:** 1 Department of Obstetrics and Gynecology, College of Medicine, Chosun University, Gwangju 501-759, Republic of Korea; 2 Department of Pathology, College of Medicine, Chosun University, Gwangju 501-759, Republic of Korea

**Keywords:** survivin, apoptosis, uterine cervix

## Abstract

Survivin is a member of the inhibitor of apoptosis protein family. Under normal circumstances, survivin is expressed in embryonic and fetal tissues, but is completely downregulated in normal adult tissues. Notably, this protein has been found to be prominently expressed in a variety of human malignant tumors. The present study was designed to evaluate the possible role of survivin in the tumorigenesis of cervical intraepithelial neoplasia and invasive squamous cell carcinoma (SCC) of the uterine cervix. In addition, it was investigated whether the nuclear or cytoplasmic expression of survivin is associated with tumor progression. In total, 71 samples of cervical squamous tissue were obtained, including 15 normal squamous epithelia, 25 high-grade squamous intraepithelial lesions (HSILs) and 31 SCCs, from cone biopsy and hysterectomy specimens and stained for survivin expression by immunohistochemistry. The intensity of survivin expression tended to increase with tumor progression (60.0% of normal mucosa, 76.0% of HSIL and 80.6% of SCC samples demonstrated high intensity survivin expression), but this correlation was not found to be statistically significant. However, a statistically significant difference was identified in the intracellular localization of survivin among the normal mucosa, HSIL and SCC samples (P<0.001). In total, 72% (18/25) of HSIL and 54.8% (17/31) of SCC cases expressed cytoplasmic staining in contrast to the nuclear staining of the normal mucosa. In addition, 64% (16/25) of HSIL and 42% (13/31) of SCC cases showed coexpression in the nucleus and cytoplasm. An inverse correlation was identified between the decrement of nuclear survivin expression and tumor progression, but was not statistically significant (P=0.08). These results indicated that analysis of the intracellular expression of survivin (particularly cytoplasmic expression) is a marker for predicting disease progression in the uterine cervix.

## Introduction

Apoptosis is an essential mechanism for the preservation of the homeostasis and morphogenesis of human tissue. Disturbance of this process by aberrantly extending cell viability or favoring accumulation of the transforming mutation is considered to contribute to carcinogenesis (1–3). The two major classes of apoptosis inhibitors are the Bcl-2 family and the inhibitor of apoptosis (IAP) protein family. The first IAP was identified in baculovirus (4) and, following this, a number of other IAPs have been identified in various mammalian species, including humans (5). Survivin is one of eight IAP proteins and has a number of distinct features which are not shared with other IAP members (6). Survivin is the shortest polypeptide, consisting of 142 amino acids, and its expression is cell cycle-regulated and occurs in the G2/M phase. In addition, survivin functions to inhibit apoptosis and regulate cell division, and also enhances angiogenesis (7,8). In general, IAP proteins are key in the negative regulation of apoptosis, and act by directly binding to caspase-3 and -7, inhibiting the process of cell death (9). The survivin protein is abundantly expressed during fetal development in humans, but rarely presents in adult tissues. However, expression of survivin has been reported in the majority human tumors, which suggests that alterations in survivin gene regulation commonly occur during tumorigenesis (10). Due to this upregulation in malignancy and its functional involvement in apoptosis as well as proliferation, survivin is currently attracting considerable interest as a potential cancer biomarker and a new target for cancer treatment (11).

Targeting the apoptosis pathways for cancer treatment is supported by several observations, which emphasize the role of aberrant apoptosis in tumorigenesis and resistance to anticancer treatment (12). Evasion of apoptosis is critical for tumor growth and is a hallmark of cancer cells (13). Specific conventional antitumor therapies, including DNA-damaging and antimicrotubule agents, exert their function by activating the intrinsic apoptosis pathway (14).

Previously, the intracellular localization of survivin in cancer cells has been reported to express biological features of cancer behavior (15). In addition, survivin mRNA levels or cytoplasmic expression of the protein has been associated with a poor outcome in various types of cancer (16–24). However, previous studies have reported opposing conclusions with regard to the significance and prognostic value of nuclear survivin expression (25–33). These observations suggest that the differential localization of survivin may indicate different protein functions and affect patient prognosis (15).

The purpose of the present study was to investigate the subcellular survivin expression levels in normal mucosa, high-grade squamous intraepithelial lesions (HSILs) and squamous cell carcinomas (SCCs) of the uterine cervix by immunohistochemistry. In addition, the association between the intracellular localization of survivin and histological diagnosis of the uterine cervix were examined, and the biological significance of the difference in intracellular localization of survivin protein was evaluated.

## Materials and methods

### Samples

In total, 71 samples of cervical squamous tissue were obtained, including 15 normal mucosa, 25 HSILs and 31 SCCs, from cone biopsy and hysterectomy performed at the Department of Obstetrics and Gynecology of the Chosun University Hospital (Gwangju, South Korea) between January, 2005 and December 2011. The Insititional Review Board of Chosun University Hospital waived the requirement for written informed consent due to the nature of the study (CHOSUN 2013-07-006-01).

### Histopathological analysis

Each specimen was re-evaluated by retrospective analysis of the medical records and the tissue slide files at the Department of Pathology, College of Medicine, Chosun University (Gwangju, South Korea). Age, human papilloma virus (HPV) infection status and histological diagnosis were assessed. The examined tissues were fixed in 10% neutral formalin and the prepared paraffin-embedded tissues were sectioned (4–5 μm in thickness). Hematoxylin and eosin staining was performed and the sections were examined under a light microscope (Olympus BX51; Olympus Corporation, Tokyo, Japan). A representative area of tumor suitable for the study purpose was selected and slides were prepared for immunohistochemical analysis.

### Immunohistochemical staining

All the specimens were tested using a rabbit anti-human survivin polyclonal antibody (1:1,000; NeoMarkers, Fremont, CA, USA), according to the manufacturer’s instructions. Immunolocalization was performed using the mouse ImmunoCruz Staining System (sc-2050; Santa Cruz Biotechnology, Inc., Santa Cruz, CA, USA), according to the manufacturer’s instructions. The staining process was performed according to standard protocol. Briefly, the 4-μm sections obtained following formalin fixation and paraffin embedding were deparaffinized in xylene and then rehydrated with distilled water through a graded series of ethanol solutions. The sections were then placed in a glass jar with 10 mmol/l citrate buffer (pH 6.0) and were irradiated in a microwave oven for 15 min. The sections were allowed to cool in the jar at room temperature for 20 min. The slides were then rinsed with Tris-buffered saline and, after quenching the endogenous peroxidase activity in 0.3% hydrogen peroxide for 10 min, a blocking reagent (sodium chloride-citrate; Ventana Medical Systems, Tucson, AZ, USA) was added for 10 min. The slides were then washed as described previously and subsequently subjected to the primary antibody reaction. Immunohistochemistry was performed on the NexES autoimmunostainer (Ventana Medical Systems) and slides were incubated with the primary antibodies for 32 min. The ultraview universial DAB detection kit (cat. no. 760–500; Ventana Medical Systems) was used as the secondary detection method. This kit includes biotinylated immunoglobulin secondary antibody, containing affinity purified goat anti-mouse IgG and IgM (b200; l g/ml) and goat anti-rabbit IgG (b200; l g/ml) in phosphate-buffered saline with preservative. Incubation was performed for 8 min and was followed by the addition of conjugated streptavidin horseradish peroxidase for 8 min. Slides were then counterstained with hematoxylin (cat. no. 760-2021; Ventana Medical Systems).

### Analysis and interpretation of staining

Representative histological sections of the lesions were immunohistochemically stained with antibody against survivin and analyzed for the expression of survivin. The immunostaining was defined as positive when >20% of tumor cells were stained for survivin in the nucleus or cytoplasm (15). The samples were subjectively classified according to the staining intensity of the nucleus and cytoplasm. Cases were classified as negative (score 0, 0–5%), weakly positive (score 1, 5–20%), moderately positive (score 3, 20–50%) and strongly positive (score 4, >50%), according to the intensity of the staining reaction (15). Next, the samples were reclassified as low intensity (score 0–2) or high intensity (scores 3 and 4).

### Statistical analysis

Statistical evaluation was performed using SPSS 12.0 (SPSS, Inc., Chicago, IL, USA). The χ^2^ test was used to demonstrate the correlation between survivin expression and histological diagnosis. P<0.05 was considered to indicate a statistically significant difference.

## Results

### Immunohistochemistry results

The expression of survivin was examined in 71 cervical lesion samples. By immunohistochemistry, survivin expression was observed in the nucleus and/or cytoplasm of cervical squamous epithelial cells. The nuclear expression of survivin without cytoplasmic expression was detected in 50.7% (36/71) of all samples [normal, 100% (15/15); HSIL, 28.0% (7/25); and SCC, 45.2% (14/31)], while the cytoplsmic expression of survivin without nuclear expression was observed in 49.3% (35/71) of all samples [normal, 0% (0/15); HSIL, 72.0% (18/25); and SCC, 54.8% (17/31)]. Furthermore, the nuclear and cytoplasmic dual reactivity of survivin was detected in 40.8% (29/71) of all squamous epithelial cell samples [normal, 0% (0/15); HSIL, 64.0% (16/25); and SCC, 41.9% (13/31)] (Table I).

### Correlation between the intensity of survivin expression and histological diagnosis

The correlation between the intensity of survivin expression and histological diagnosis was examined. The intensity of survivin expression tended to increase with tumor progression; 60.0% of normal mucosa, 76.0% of HSILs and 80.6% of SCCs revealed high intensity of survivin expression. However, this correlation was not found to be statistically significant (Table II).

### Intracellular localization of survivin among normal mucosa, HSIL and SCC samples

**Figure 1:**
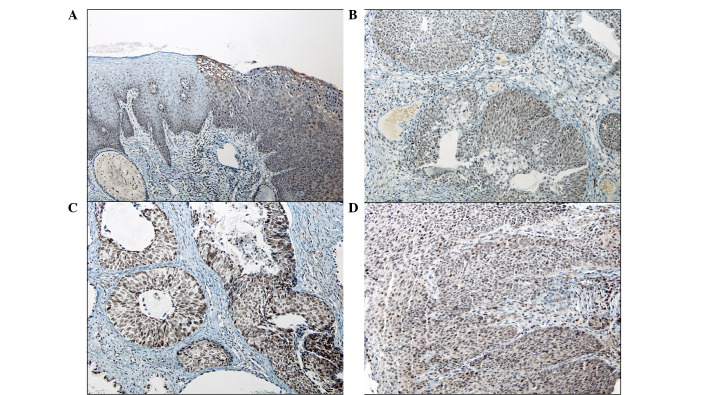
Immunohistochemical staining of survivin. (A) High-grade squamous intraepithelial lesion (cervical intraepithelial lesion 2, upper right) with viral cytopathic effects associated with human papillomavirus infection shows strong cytoplasmic immunoreactivity in contrast to the adjacent normal squamous epithelium. Invasive squamous cell carcinoma exhibits variable survivin expression, including (B) weak nuclear and moderate cytoplasmic expression, (C) strong nuclear expression without cytoplasmic expression and (D) strong nuclear and weak cytoplasmic expression.

In viral cytopathic lesions (with HPV cytopathic effects), the koilocytes showed cytoplasmic staining without nuclear expression. A statistically significant difference was identified in the intracellular localization of survivin among the normal mucosa, HSILs and SCCs (P<0.001; Table I). In total, 72.0% (18/25) of HSIL and 54.8% (17/31) of SCC cases expressed cytoplasmic immunoreactivity, in contrast to the nuclear staining of all normal mucosa samples. In addition, 64.0% (16/25) of HSIL and 41.9% (13/31) of SCC cases showed coexpression in the nucleus and cytoplasm (Fig. 1A–D). An inverse correlation was identified between the decrement of nuclear survivin expression and tumor progression, but this was not statistically significant (P=0.08).

## Discussion

In a number of developing countries, cervical cancer is the most common fatal malignancy in females; however, there has been a marked reduction in mortalities due to cervical cancer as a result of the success of diagnostic cytopathology (34–36). The majority of cervical cancers are SCC and most cervical SCC cases are preceded by cervical intraepithelial lesions (CINs), including low-grade squamous intraepithelial lesions (LSILs) and HSILs (37,38). While HSIL more commonly appears to progress to invasive cancer compared with LSIL, it is not always possible to determine the risk of progression in individual SILs (39). Molecular markers of malignant potential may be important in the detection of lesions that exhibit the greatest potential for progression to cancer and may also be involved in increasing the sensitivity of current diagnostic techniques (39).

Previously, survivin mRNA levels or cytoplasmic protein expression have been associated with poor outcome in various types of cancer, including breast cancer (16), lymphoma (17), non-small cell lung cancer (18), liver cancer (19), gastric carcinoma (20), ovarian carcinoma (24) and colorectal cancer (21–23). In the present study, the cytoplasmic expression of survivin was found to increase in dysplastic lesions (HSILs and SCCs) compared with the normal mucosa. Certain previous contradictory studies have shown that the nuclear staining of survivin is associated with a favorable prognosis in gastric (25) and breast (26) cancer. In addition, the expression of nuclear survivin in osteosarcoma (27), transitional cell carcinoma of the urinary bladder (28), pancreatic cancer (29) and non-small cell lung cancer (30) has been found to correlate with a good prognosis. However, in hepatocellular carcinoma, esophageal SCC and epithelial ovarian tumors, the expression of nuclear survivin has been found to correlate with an unfavorable prognosis (31–33). In the present study, an inverse correlation was identified between the decrement of nuclear survivin expression and tumor progression, but was not statistically significant (P=0.08). The reason for these different prognostic results in the subcellular localization of survivin in different cancers remains unclear (15). However, despite certain inconsistent results, the bulk of data concerning numerous human cancer types support the theory that the cytoplasmic expression of survivin is associated with cancer progression and poor prognosis (9,40).

In the present study, survivin was detected in the normal squamous epithelia, cervical dysplasia (HSILs and SCCs *in situ*) and invasive SCC. Although there is an increasing tendency of disease progression, the intensity of survivin expression is unlikely to have a potential role as a diagnostic or prognostic marker for cervical SCC. These results suggest that the biological behavior of cervical dysplasia may differ according to the intracellular localization of survivin. The function of cytoplasmic survivin may be important for malignant progression. Elucidation of the role of cytoplasmic survivin may define the clinical significance of survivin expression in cervical dysplastic lesions. In addition, we suggest that the inhibition of the cytoplasmic localization of survivin may present a novel strategy for cervical cancer treatment.

## Figures and Tables

**Table I:** Differences between the intracellular localization of survivin in the normal mucosa, HSIL and SCC (%).

Localization	Normal mucosa, n (%)	HSIL, n (%)	SCC, n (%)	Total, n (%)	P-value
Nucleus	15 (100)	7 (28.0)	14 (45.2)	36 (50.7)	
Cytoplasm	0 (0)	18 (72.0)	17 (54.8)	35 (49.3)	
Dual^a^	0 (0)	16 (64.0)	13 (41.9)	29 (40.8)	<0.001^b^
Total	15 (21.1)	25 (35.2)	31 (43.7)	71 (100.0)	

a Localization to the nucleus and cytoplasm;

b P<0.05 was considered to indicate a stastically significant difference.

HSIL, high-grade squamous intraepithelial lesion; SCC, squamous cell carcinoma.

**Table II:** Differences between the intensity of survivin expression in the normal mucosa, HSIL and SCC (%).

Intensity	Normal mucosa, n (%)	HSIL, n (%)	SCC, n (%)	Total, n (%)	P-value
Low^a^	6 (40.0)	6 (24.0)	6 (19.4)	18 (25.4)	
High^b^	9 (60.0)	19 (76.0)	25 (80.6)	53 (74.6)	<0.1^c^
Total	15 (21.1)	25 (35.2)	31 (43.7)	71 (100.0)	

Staining intensity scores:

a low, scores 0 and 1; and

b high, scores 2 and 3.

c P<0.05 was considered to indicate a statistically significant difference.

HSIL, high-grade squamous intraepithelial lesion; SCC, squamous cell carcinoma.

## References

[1] Thompson CB (1995). Apoptosis in the pathogenesis and treatment of disease. Science.

[2] Barinaga M (1998). Death by dozens of cuts. Science.

[3] Kiechle FL, Zhang X (2002). Apoptosis: biochemical aspects and clinical implications. Clin Chim Acta.

[4] Crook NE, Clem RJ, Miller LK (1993). An apoptosis-inhibiting baculovirus gene with a zinc finger-like motif. J Virol.

[5] LaCasse EC, Baird S, Korneluk RG, MacKenzie AE (1998). The inhibitors of apoptosis (IAPs) and their emerging role in cancer. Oncogene.

[6] Adamkov M, Výbohová D, Horáček J, Kovalská M, Furjelová M (2013). Survivin expression in breast lobular carcinoma: correlations with normal breast tissue and clinicomorphological parameters. Acta Histochem.

[7] Li F, Yang J, Ramnath N, Javle MM, Tan D (2005). Nuclear or cytoplasmic expression of survivin: what is the significance?. Int J Cancer.

[8] Li F, Brattain MG (2006). Role of the survivin gene in pathophysiology. Am J Pathol.

[9] Lee WS, Cho SB, Rew JS, Lee JH, Park CS, Joo YE (2009). Expression of survivin in gastric carcinoma and its relation to tumor cell proliferation and apoptosis. Kor J Pathol.

[10] Ambrosini G, Adida C, Altieri DC (1997). A novel anti-apoptotic gene, survivin, expressed in cancer and lymphoma. Nat Med.

[11] Altieri DC (2008). Survivin, cancer networks and pathway-directed drug discovery. Nat Rev Cancer.

[12] Mita AC, Mita MM, Nawrocki ST, Giles FJ (2008). Survivin: key regulator of mitosis and apoptosis and novel target for cancer therapeutics. Clin Cancer Res.

[13] Hanahan D, Weinberg RA (2000). The hallmarks of cancer. Cell.

[14] Adams JM, Cory S (2001). Life-or-death decisions by the Bcl-2 protein family. Trends Biochem Sci.

[15] Qi G, Tuncel H, Aoki E, Tanaka S, Oka S, Kaneko I (2009). Intracellular localization of survivin determines biological behavior in colorectal cancer. Oncol Rep.

[16] Tanaka K, Iwamoto S, Gon G, Nohara T, Iwamoto M, Tanigawa N (2000). Expression of survivin and its relationship to loss of apoptosis in breast carcinomas. Clin Cancer Res.

[17] Adida C, Haioun C, Gaulard P, Lepage E, Morel P, Briere J (2000). Prognostic significance of survivin expression in diffuse large B-cell lymphomas. Blood.

[18] Monzó M, Rosell R, Felip E, Astudillo J, Sánchez JJ, Maestre J (1999). A novel anti-apoptosis gene: Re-expression of survivin messenger RNA as a prognosis marker in non-small-cell lung cancers. J Clin Onol.

[19] Ikeguchi M, Ueta T, Yamane Y, Hirooka Y, Kaibara N (2002). Inducible nitric oxide synthase and survivin messenger RNA expression in hepatocellular carcinoma. Clin Cancer Res.

[20] Wakana Y, Kasuya K, Katayanagi S, Tsuchida A, Aoki T, Koyanagi Y (2002). Effect of survivin on cell proliferation and apoptosis in gastric cancer. Oncol Rep.

[21] Kawasaki H, Altieri DC, Lu CD, Toyoda M, Tenjo T, Tanigawa N (1998). Inhibition of apoptosis by survivin predicts shorter survival rates in colorectal cancer. Cancer Res.

[22] Sarela AI, Macadam RC, Farmery SM, Markham AF, Guillou PJ (2000). Expression of the antiapoptosis gene, survivin, predicts death from recurrent colorectal carcinoma. Gut.

[23] Sarela AI, Scott N, Ramsdale J, Markham AF, Guillou PJ (2001). Immunohistochemical detection of the anti-apoptosis protein, survivin, predicts survival after curative resection of stage II colorectal carcinomas. Ann Surg Oncol.

[24] Sui L, Dong Y, Ohno M, Watanabe Y, Sugimoto K, Tokuda M (2002). Survivin expression and its correlation with cell proliferation and prognosis in epithelial ovarian tumors. Int J Oncol.

[25] Okada E, Murai Y, Matsui K, Isizawa S, Cheng C, Masuda M, Takano Y (2001). Survivin expression in tumor cell nuclei is predictive of a favorable prognosis in gastric cancer patients. Cancer Lett.

[26] Kennedy SM, O’Driscoll L, Purcell R, Fitz-Simons N, McDermott EW, Hill AD (2003). Prognostic importance of Survivin in breast cancer. Br J Cancer.

[27] Trieb K, Lehner R, Stulnig T, Sulzbacher I, Shroyer KR (2003). Survivin expression in human osteosarcoma is a marker for survival. Eur J Surg Oncol.

[28] Lehner R, Lucia MS, Jarboe EA, Orlicky D, Shroyer AL, McGregor JA, Shroyer KR (2002). Immunohistochemical localization of the IAP protein survivin in bladder mucosa and transitional cell carcinoma. Appl Immunohistochem Mol Morphol.

[29] Tonini G, Vincenzi B, Santini D (2005). Nuclear and cytoplasmic expression of survivin in 67 surgically resected pancreatic cancer patients. Br J Cancer.

[30] Vischioni B, Van der Valk P, Span SW, Kruyt FA, Rodriguez JA, Giaccone G (2004). Nuclear localization of survivin is a positive prognostic factor for survival in advanced non-small-cell lungcancer. Ann Oncol.

[31] Ito T, Shiraki K, Sugimoto K, Yamanaka T, Fujikawa K, Ito M (2000). Survivin promotes cell proliferation in human hepatocellular carcinoma. Hepatology.

[32] Grabowski P, Kühnel T, Mühr-Wilkenshoff F, Heine B, Stein H, Höpfner M (2003). Prognostic value of nuclear survivin expression in oesophageal squamous cell carcinoma. Br J Cancer.

[33] Tringler B, Lehner R, Shroyer AL, Shroyer KR (2004). Immunohistochemical localization of survivin in serous tumors of the ovary. Appl Immunohistochem Mol Morphol.

[34] Birley HD (1995). Human papillomaviruses, cervical cancer and the developing world. Ann Trop Med Parasitol.

[35] American Cancer Society (1999). Cancer Facts and Figures 1999.

[36] Walboomers JM, Jacobs MV, Manos MM, Bosch FX, Kummer JA, Shah KV (1999). Human papillomavirus is a necessary cause of invasive cervical cancer worldwide. J Pathol.

[37] Richart RM (1973). Cervical intraepithelial neoplasia. Pathol Annu.

[38] Wright TC, Kurman RJ, Ferenczy A, Kurman RJ (1994). Precancerous lesions of the cervix. Blaustein’s Pathology of the Female Genital Tract.

[39] Frost M, Jarboe EA, Orlicky D, Gianani R, Thompson LC, Enomoto T, Shroyer KR (2002). Immunohistochemical localization of survivin in benign cervical mucosa, cervical dysplasia, and invasive squamous cell carcinoma. Am J Clin Pathol.

[40] Xie D, Zeng YX, Wang HJ, Wen JM, Tao Y, Sham JS, Guan XY (2006). Expression of cytoplasmic and nuclear Survivin in primary and secondary human glioblastoma. Br J Cancer.

